# Establishment and Characterization of an Immortalized Oviduct Epithelial Cell Line from Yak (*Bos grunniens*)

**DOI:** 10.3390/ani15172509

**Published:** 2025-08-26

**Authors:** Wenyi Cai, Yuting Jiang, Xuelian Xu, Qiliang Ma, Congcong Xu, Wei Fu, Daoliang Lan

**Affiliations:** 1 College of Animal & Verterinary Sciences, Southwest Minzu University, Chengdu 610041, China; 220905001004@stu.swun.edu.cn (W.C.);; 2 Key Laboratory of Qinghai-Tibetan Plateau Animal Genetic Resource Reservation and Utilization of Ministry of Education, Southwest Minzu University, Chengdu 610041, China

**Keywords:** yak, oviduct epithelial cells, immortalization, hTERT, SV40LT

## Abstract

Oviduct epithelial cells (OECs) constitute a critical component of the oviductal mucosa, providing essential microenvironmental support for fertilization and early embryonic development. Their frequent application in embryo co-culture systems is constrained in yaks (*Bos grunniens*) by limited tissue availability and the short lifespan of primary yak oviduct epithelial cells (YOECs). To address this limitation, we established immortalized YOEC lines using a lentiviral vector system. Primary YOECs isolated from reproductive tract tissues of adult female yaks via enzymatic digestion were immortalized through individual and combined transfection with simian virus 40 large T antigen (SV40LT) and human telomerase reverse transcriptase (hTERT). The resulting immortalized lines (YOECs-S: SV40LT alone; YOECs-HS: dual SV40LT/hTERT) kept their typical cobblestone shape and still made cytokeratin 18. Both lines exhibited stable SV40LT and hTERT expression (*p* > 50), maintained diploid karyotypes, and demonstrated serum-dependent growth, contact inhibition, and hormone responsiveness. Notably, YOECs-HS displayed superior proliferative capacity and phenotypic stability during long-term culture. This study reports the first successful establishment and comprehensive characterization of immortalized YOEC lines. These validated models provide a valuable experimental platform for optimizing yak embryo–oviduct epithelial cell co-culture systems and advancing reproductive research in this high-altitude-adapted species.

## Simple Summary

Oviduct epithelial cells (OECs), as critical components of the oviductal mucosa, play essential roles in reproductive physiology. This study established primary yak oviduct epithelial cell (YOEC) cultures and generated immortalized YOEC lines through lentiviral-mediated expression of simian virus 40 large T antigen (SV40LT) and human telomerase reverse transcriptase (hTERT). Comparative assessment of immortalization efficiency revealed that both single transfection (SV40LT alone: YOECs-S) and dual transfection (SV40LT + hTERT: YOECs-HS) successfully yielded immortalized lines. These cell lines have maintained stable proliferation through >50 passages, providing validated in vitro models for optimizing yak embryo–oviduct epithelial cell co-culture systems.

## 1. Introduction

The yak (*Bos grunniens*) is a species endemic to the Qinghai–Tibet Plateau, functioning as both an ecosystem engineer and an economic cornerstone [1]. However, genetic degradation and inbreeding within its populations pose significant threats to its long-term sustainability [2]. To address these challenges, in vitro embryo production (IVP) coupled with embryo transplantation represents a highly promising strategy [3,4,5]. The success of IVP depends critically not only on the quality of oocytes and spermatozoa but also on the in vitro culture environment. This environment must meticulously replicate the in vivo microenvironment of natural embryonic development [6].

The fallopian tube constitutes the core of this in vivo microenvironment. It actively coordinates early embryonic development rather than merely serving as an anatomical conduit between the ovary and uterus [7]. Mammalian oviductal epithelial cells (OECs) dynamically protect the embryo through specialized secretions and signaling pathways [8,9,10]; these functions are inadequately replicated in conventional in vitro fertilization (IVF) systems. This biological necessity is well-demonstrated in conventional cattle (Bos taurus), where co-culture with bovine oviductal epithelial cells (BOECs) significantly enhances the developmental kinetics and quality of IVP embryos [11]. Reflecting these benefits, the use of IVF is expanding rapidly in livestock breeding and endangered-species programs, and OEC-assisted cultures are increasingly incorporated to maximize embryo quality and pregnancy outcomes [12,13]. Primary OECs exhibit limited proliferative capacity in vitro, typically lasting fewer than 10 passages. This constraint impedes sustained research applications.

Current technology enables cell immortalization through the modulation of telomerase activation, tumor suppressor gene inactivation, and oncogene expression [14,15,16]. Immortalized OEC lines have been established for humans [17], poultry [18], and cattle [19]. These facilitate continuous research into embryo–maternal communication and the development of improved IVF culture media. However, no such tools exist for the high-altitude-adapted yak. Yak reproductive physiology is unique, governing embryonic development under conditions of low pressure and hypoxia [20]. This distinctiveness may necessitate different immortalization strategies compared to the conventional bovine model. Consequently, establishing a yak-specific immortalization protocol for yak oviductal epithelial cells (YOECs) constitutes a critical research priority.

This study aimed to establish and characterize immortalized yak oviduct epithelial cell (YOEC) lines. Two distinct immortalized cell lines—YOECs-S (expressing SV40 large T antigen) and YOECs-HS (co-expressing hTERT and SV40LT)—were generated via lentiviral transduction. These lines underwent comprehensive biological characterization in accordance with internationally recognized standards for immortalized cells (ICLAC guidelines). This foundational work enables the optimization of embryo–oviduct co-culture systems, investigation of reproductive system-specific behaviors in immortalized cells, and the advancement of yak reproductive biotechnology applications.

## 2. Materials and Methods

### 2.1. Experimental Animals

Oviducts were aseptically collected from healthy adult female yaks at a Jinchuan County (Aba Prefecture) slaughterhouse. Tissues underwent sequential processing comprising initial irrigation with sterile physiological saline, immersion in PBS containing 1% penicillin–streptomycin, and storage in refrigerated containers at 4 °C. All specimens were transported to the laboratory within 72 h post-collection under continuous temperature monitoring.

### 2.2. Isolation and Primary Culture of YOECs

Oviducts were dissected free of adherent connective tissue, and underwent surface decontamination with 75% ethanol and PBS containing 1% penicillin–streptomycin. Mucosal epithelium was scraped using a scalpel blade (45° angle) into 15 mL conical tubes containing 4 mL antibiotic-supplemented PBS. After centrifugation (500× *g*, 5 min), pellets were digested in 0.2% collagenase I (ThermoFisher, 17100017, New York, NY, USA) at 37 °C for 2 h with agitation. Digestion completeness was confirmed by macroscopic absence of tissue fragments. Enzymatic activity was neutralized with equal-volume DMEM/F12 (ThermoFisher, 11320033, Grand Island, NY, USA) supplemented with 10% FBS (Sbjbio, BC-SE-FBS07, Nanjing, China). Cell suspensions were filtered through 40 μm strainers, centrifuged (500× *g*, 5 min), and resuspended in complete medium (DMEM/F12 + 10% FBS + 1% Antibiotic–Antimycotic). Primary cultures were maintained at 37 °C/5% CO_2_.

### 2.3. Passaging and Cryopreservation of YOECs

Confluent monolayers (80%) were subjected to two washes with PBS followed by dissociation in 0.25% trypsin with EDTA (ThermoFisher, 25200056, Grand Island, NY, USA) for 4 min at 37 °C. Enzyme activity was neutralized with complete medium upon confirmation of cellular rounding via microscopy. Mechanical disaggregation was achieved through pipetting, and resulting suspensions were centrifuged at 500× *g* for 5 min. Resuspended cells were subcultured at 1:3 ratios. For cryopreservation, cells were stored at a density of 1 × 10^6^ cells/mL in cryoprotectant solution (1 mL/vial). The cryoprotective solution consisted of 10% DMSO (BOSTER, YG0040, Wuhan, China) and 90% serum. The cryovials were placed into a specialized freezing container and maintained at −80 °C for 24 h, and ultimately transferred to liquid nitrogen vapor phase for long-term archival storage.

### 2.4. Establishment of Immortalized YOEC Lines

The current standard methodology for establishing immortalized cell lines primarily relies on transfection with Simian Virus 40 Large T antigen (SV40T) and/or Human Telomerase Reverse Transcriptase (hTERT). Using the laboratory’s existing plasmids pBABE-puro-hTERT and pBABE-puro-SV40LT as templates, the hTERT (3399 bp) and SV40LT (2127 bp) fragments were amplified via PCR with specific primers. These fragments were then cloned into the pLVX-Puro vector backbone, resulting in the construction of the pLVX-hTERT-Puro and pLVX-SV40LT-Puro plasmids, respectively. Subsequently, pLVX-hTERT-Puro and pLVX-SV40LT-Puro were co-transfected into 293T cells alongside the lentiviral packaging plasmids psPAX2 and pMD2.G using Lipofectamine 2000 (ThermoFisher, 11668019, Carlsbad, CA, USA) to generate lentiviral particles. The viral supernatants harvested at both 48 and 72 h post-transfection were concentrated (Solarbio, CA2320, Beijing, China).

YOES cells at passage 3 (P3) in good growth condition were seeded into 6-well plates. When cells reached 50% confluency, lentiviral transduction was performed. For each well, 10 μL of the following viral supernatants were added separately: hTERT lentivirus, SV40LT lentivirus, or combined hTERT/SV40LT lentivirus. Following 4 h of incubation, Polybrene (Solarbio, H8761, Beijing, China) was supplemented to a final concentration of 8 μg/mL per well. At 24 h post-transduction, the medium was replaced with fresh complete medium containing 1.5 μg/mL puromycin (MCE, HY-B1743, Monmouth Junction, NJ, USA). EGFP expression was assessed by fluorescence microscopy (Olympus, IX73, Tokyo, Japan) at 72 h. Surviving cell colonies were subjected to monoclonal isolation via limiting dilution in 96-well plates. Positively selected monoclonal colonies were subsequently expanded for further culture.

### 2.5. Hematoxylin and Eosin (H&E) Staining Identification

Cells seeded on coverslips in 12-well plates at 1 × 10^5^ cells/mL were allowed to adhere for 24 h. After two washes with phosphate-buffered saline (PBS), samples were fixed in 4% paraformaldehyde at room temperature for 15 min. Cells were then stained with hematoxylin solution (Solarbio, G1120, Beijing, China) for 10 min, followed by a 3 min wash under running tap water. Differentiation was performed in 1% acid–alcohol for 2 sec, after which samples were rinsed with tap water for 1 min. Subsequent dehydration through graded ethanol series and mounting with synthetic resin enabled bright-field microscopic analysis (Carl Zeiss AG, Primo Star, Oberkochen, Germany).

### 2.6. Immunofluorescence (IF) Identification

Cells cultured on coverslips in 12-well plates underwent sequential immunofluorescence processing; three 3 min PBS washes, fixation in 4% paraformaldehyde (RT, 30 min), permeabilization with 0.5% Triton X-100 (Beyotime, ST1723, Shanghai, China) (15 min), blocking in 3% BSA (1 h), and incubation with primary antibodies [CK-18 (rabbit polyclonal, 1:200, Bioss, bs-1339R, Beijing, China), Vimentin (mouse monoclonal, 1:200, Boster, BM0135, Wuhan, China)] overnight at 4 °C. Following three PBS washes, samples were incubated with goat anti-rabbit IgG secondary antibody (1:250, Boster, BA1055, Wuhan, China) for 2 h in darkness, counterstained with DAPI (5 min) (Boster, AR1176, Wuhan, China), mounted in ProLong^®^ Glass Antifade Mountant (ThermoFisher, P36984, Eugene, OR, USA), and imaged using fluorescence microscopy.

### 2.7. Cell Viability Assay (CCK-8)

Cell viability was assessed via CCK-8 assay in passage 3 (P3) primary YOECs, passage 30 (P30) YOECs-S, and P30 YOECs-HS seeded at 2000 cells/well in 96-well plates (*n* = 8 groups × 3 replicates). Every 24 h for 8 consecutive days, selected wells received 10 μL CCK-8 reagent (Vazyme, A311-01, Nanjing, China), incubated at 37 °C for 2 h in darkness, with absorbance measured at 450 nm using a microplate reader(BIO-RAD, iMark, Hercules, CA, USA). Growth curves were generated by plotting optical density against time.

### 2.8. qPCR and PCR Analysis

Total RNA was extracted from YOECs, immortalized YOECs-S, YOECs-HS, HeLa, and 293T cell lines using TRIzol™ reagent (VazyMe, R411, Nanjing, China), followed by reverse transcription into cDNA (Beyotime, D7168S, Shanghai, China). Gene-specific primers (Table 1) were designed and synthesized for qPCR analysis (Roche, LightCycler 96, Basel, Switzerland) of hTERT and SV40LT transgene stability, while conventional PCR assessed estrogen receptor (ER) and progesterone receptor (PR) expression profiles.

### 2.9. Western Blot (WB) Detection

Protein lysates from YOECs, immortalized YOECs-S, YOECs-HS, HeLa, and 293T cells were extracted using RIPA buffer (Beyotime, P0013B, Shanghai, China). Following separation via SDS-PAGE (10% resolving gel) (Yamei, PG112, Shanghai, China), proteins were transferred to PVDF membranes and blocked with 5% non-fat milk for 1 h at room temperature. Membranes were incubated overnight at 4 °C with primary antibodies against hTERT (1:1000, Bioss, bs-0233R, Beijing, China), SV40LT (1:1000, Sangon, D290001, Shanghai, China), and β-actin (1:5000, Bioss, bsm-33032R, Beijing, China) as loading control. After TBST washes, horseradish peroxidase-conjugated secondary antibodies (1:20,000, Beyotime, A0208, Shanghai, China) were applied for 1 h. Protein bands were visualized using enhanced chemiluminescence (Beyotime, P0018S, Shanghai, China) with image capture via a chemical imaging system (Invitrogen, iBright CL1000, Carlsbad, CA, USA) under standardized exposure conditions.

### 2.10. Karyotype Analysis

Chromosomal analysis was performed on P3 primary YOECs, P30 immortalized YOECs-S, and P30 YOECs-HS cells. Metaphase arrest was induced by 4 h treatment with 0.07 μg/mL colchicine. Following trypsinization, cells were subjected to hypotonic shock in 8 mL 0.075 M KCl (37 °C, 15 min), then fixed through three cycles of centrifugation (500× *g*, 8 min) and resuspension in freshly prepared Carnoy’s fixative (3:1 methanol/glacial acetic acid). Chromosome spreads were prepared by dropping cell suspensions onto pre-chilled slides from 15 cm height, flame dried, aged at room temperature for 96 h, and stained with 5% Giemsa solution (Beyotime, C0131, Shanghai, China) (15 min). Karyotypes were examined under oil immersion microscopy (100× objective) with ≥30 metaphase spreads analyzed per cell line.

### 2.11. Serum Dependency Analysis

Serum dependence was assessed in P3 primary YOECs, P30 YOECs-S, and P30 YOECs-HS cells seeded at 1 × 10^4^ cells/well in 96-well plates (quadruplicate wells per condition). Cells were maintained in media containing 0%, 10%, or 20% fetal bovine serum (FBS). After 72 h incubation, 10 μL CCK-8 reagent (Vazyme, A311-01, Nanjing, China) was added per well followed by 2 h dark incubation at 37 °C. Absorbance at 450 nm was quantified using a microplate reader to determine proliferation indices.

### 2.12. Statistical Analysis

All experiments comprised three independent biological replicates. Fluorescence signal quantification was performed using ImageJ (version 2.0, Bethesda, MD, USA). Statistical significance was determined in SPSS (version 20.0, IBM, Chicago, IL, USA), where *p*-value < 0.05 indicated significant differences. Data visualization was executed in GraphPad Prism (version 8.0, San Diego, CA, USA).

## 3. Results

### 3.1. Isolation and Culture of YOECs

**Figure 1:**
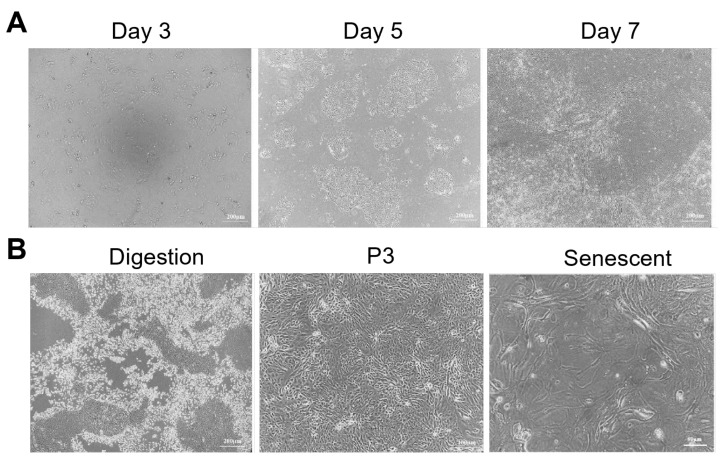
Primary YOECs isolation and senescence morphology. (**A**) Temporal progression of YOECs in vitro: day 3 (initial clustering); day 5 (epithelial sheet formation); day 7 (confluent monolayer) (40×, Scale bar indicates 200 µm). (**B**) Senescent phenotypes at late passage (P10): difficulty in trypsin digestion (40×, Scale bar indicates 200 µm); reduced cell density (100×, Scale bar indicates 100 µm); and cytoplasmic vacuolation (200×, Scale bar indicates 50 µm).

YOECs were successfully isolated through mechanical scraping followed by 0.2% collagenase I digestion (37 °C, 2 h), optimizing cellular yield and viability while accommodating tissue density constraints. Isolated cells exhibited characteristic cobblestone morphology with progressive growth patterns; initial cluster formation at day 3, epithelial sheet development by day 5 (Figure 1A), and confluent monolayers with defined cell borders by day 7. While P3 YOECs maintained stable cobblestone phenotypes, primary cultures demonstrated limited proliferative capacity, undergoing senescence by passage 10 (P10). Late-passage cells displayed reduced mitotic activity and prominent cytoplasmic vacuolation (Figure 1B).

### 3.2. Identification of YOECs

**Figure 2:**
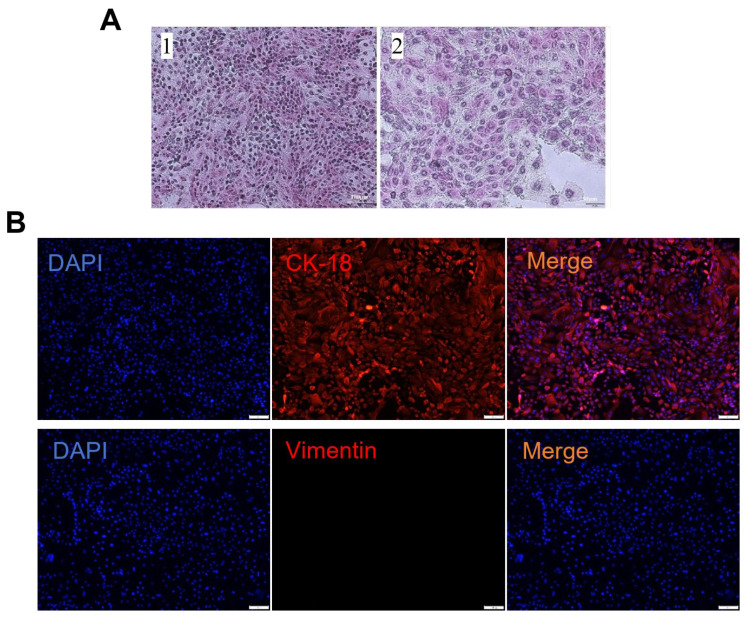
Histomorphological and immunofluorescence characterization of primary YOECs. (**A**) H&E staining showing epithelial morphology: (A1) Confluent monolayer (100×, Scale bar indicates 100 µm); (A2) Nuclear–cytoplasmic details (200×, Scale bar indicates 50 µm). (**B**) Immunophenotyping: CK-18^+^/vimentin^−^ staining confirming epithelial purity (100×, Scale bar indicates 100 µm).

Histological examination via H&E staining confirmed characteristic epithelial morphology in YOECs, displaying polygonal cells arranged in cobblestone monolayers with abundant cytoplasm and large, rounded nuclei (Figure 2A). Immunofluorescence analysis further validated epithelial identity, demonstrating positive cytokeratin 18 (CK-18) expression and negative vimentin staining. This marker profile confirms isolation of high-purity oviduct epithelial cells devoid of mesenchymal contamination (Figure 2B).

### 3.3. Establishment of Immortalized YOECs

**Figure 3:**
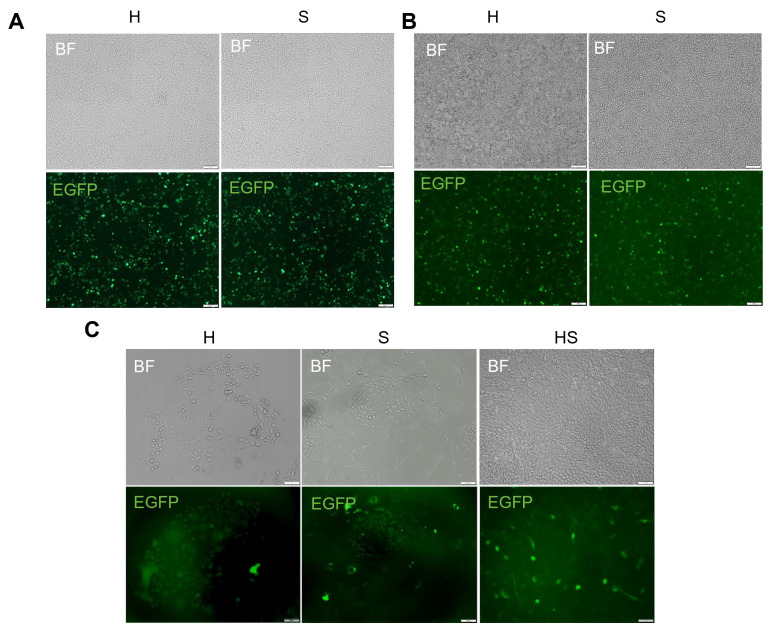
Lentiviral packaging and transduction efficiency. (**A**) 293T cells 72 h post-transfection with packaging plasmids (100×, Scale bar indicates 100 µm). (**B**) Transduction validation in 293T cells (72 h post-infection) (100×). (**C**) Comparative fluorescence in YOECs transduced with hTERT-only, SV40LT-only, or dual hTERT/SV40LT constructs (72 h post-infection) (100×, Scale bar indicates 100 µm). H, hTERT-only; S, SV40LT-only; HS, dual hTERT/SV40LT.

Lentiviral particles expressing hTERT or SV40LT were generated by co-transfecting pLVX-hTERT-Puro and pLVX-SV40LT-Puro with packaging plasmids (psPAX2/pMD2.G) into 293T cells. Peak fluorescence intensity occurred at 72 h post-transfection (Figure 3A), indicating optimal viral packaging. Harvested supernatants demonstrated functional transduction capacity when applied to 293T cells (Figure 3B). Following YOECs transduction with concentrated virus, differential fluorescence intensity was observed at 72 h; co-transduction (hTERT + SV40LT) yielded a significantly stronger signal than single-gene transduction (Figure 3C). Puromycin-resistant colonies underwent monoclonal isolation and expansion for subsequent characterization.

### 3.4. Morphological Identification and Growth Characteristics of Immortalized YOECs

**Figure 4:**
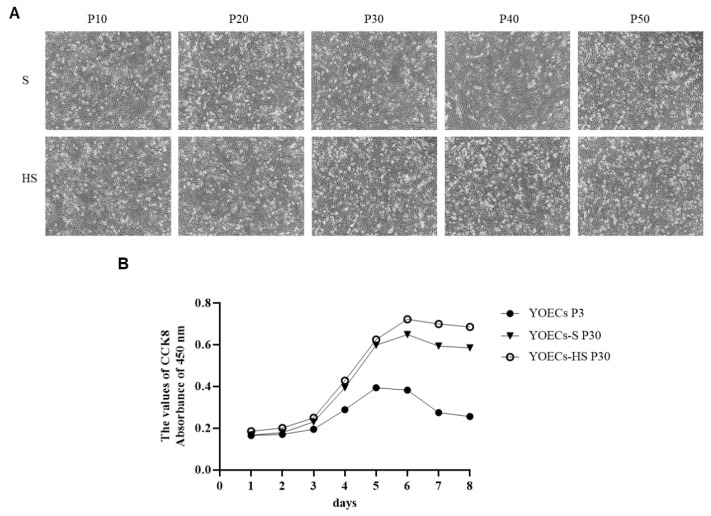
Characterization of immortalized YOECs. (**A**) Morphology of YOECs-S and YOECs-HS cells at indicated passages (100×, Scale bar indicates 100 µm). (**B**) Proliferation kinetics of YOECs-S and YOECs-HS cells at P30 compared to primary YOECs, assessed by CCK-8 assay. Data represent mean ± SD (n = 3 replicates per group per time point).

Morphological and growth characteristics of immortalized YOECs were assessed across multiple passages. YOECs-H cells exhibited significantly reduced proliferation rates and prominent vacuolization by P10. In contrast, both YOECs-S and YOECs-HS cells demonstrated sustained robust proliferation and stable growth phenotypes, achieving successful serial passaging beyond P50 (Figure 4A). Morphologically, immortalized YOECs-S and YOECs-HS displayed a reduced cell volume compared to primary YOECs [13], adopting a characteristic cobblestone-like morphology with tightly packed arrangements. Furthermore, these immortalized lines demonstrated increased sensitivity to temperature fluctuations with progressive passaging, frequently resulting in cell rounding and detachment under suboptimal low-temperature conditions.

Assessment of proliferative capacity using the CCK-8 assay revealed significantly higher viability in YOECs-S and YOECs-HS cells relative to primary YOECs (*p* < 0.05). All three cell populations exhibited similar growth kinetics: an initial lag phase (days 1–3), followed by exponential growth (days 4–5), and progression into the stationary phase by day 6. Notably, at matched passage numbers (P30), YOECs-HS cells displayed a modest but consistent increase in proliferative activity compared to YOECs-S cells (Figure 4B).

### 3.5. Stable Expression and Protein Distribution of hTERT and SV40LT in Immortalized YOECs

**Figure 5:**
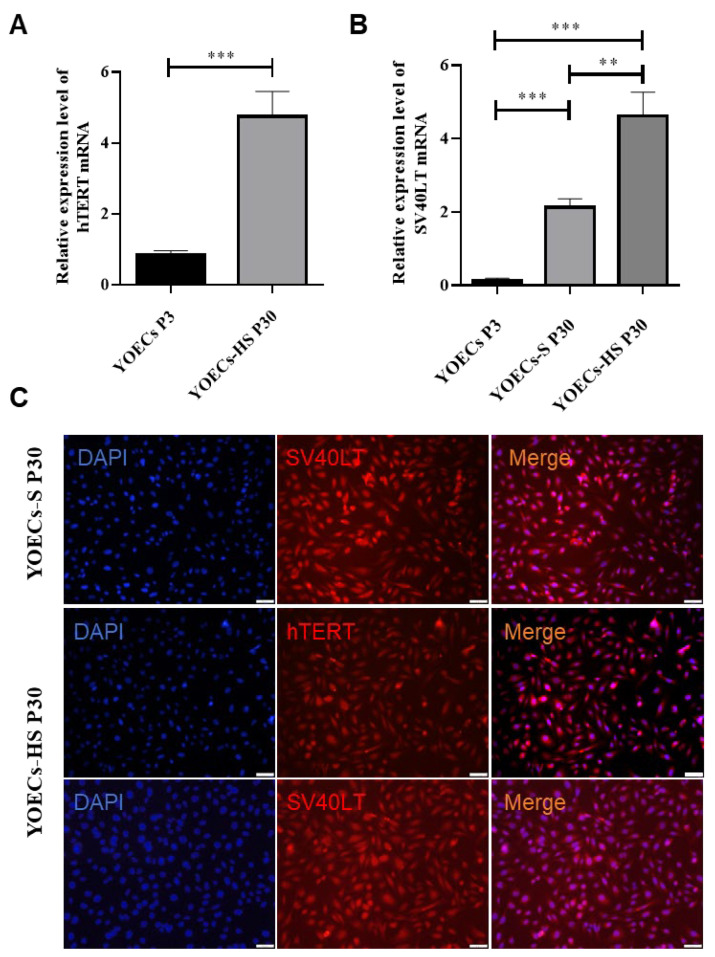
Expression and localization of hTERT and SV40LT in immortalized YOECs. (**A**) hTERT mRNA expression levels in YOECs-HS cells compared to primary YOECs (P3). (**B**) SV40LT mRNA expression levels in YOECs-S and YOECs-HS cells (P30) compared to primary YOECs (P3). Data represent mean ± SD. ** *p* < 0.01, *** *p* < 0.001. (**C**) Subcellular localization of SV40LT protein (green) in P30 YOECs-S and YOECs-HS cells. Nuclei were counterstained with DAPI (blue) (200×, Scale bar indicates 50 µm).

Stable expression and subcellular localization of hTERT and SV40LT were evaluated in immortalized YOECs. Quantitative PCR (qPCR) analysis comparing P30 YOECs-S and YOECs-HS cells with P3 primary YOECs revealed significantly elevated mRNA expression levels of both hTERT and SV40LT in P30 YOECs-HS cells relative to primary controls. Furthermore, SV40LT mRNA expression in P30 YOECs-HS cells was significantly higher than in P30 YOECs-S cells. Notably, SV40LT mRNA expression was nearly undetectable in P3 primary YOECs (Figure 5A,B).

Immunofluorescence staining confirmed the presence of SV40LT protein in both the cytoplasmic and nuclear compartments of YOECs-S and YOECs-HS cells (Figure 5C). In contrast, negligible expression of hTERT and SV40LT proteins was observed in P3 primary YOECs, where only DAPI nuclear counterstaining was evident.

### 3.6. Stable Expression of hTERT and SV40LT Proteins in Immortalized YOECs

**Figure 6:**
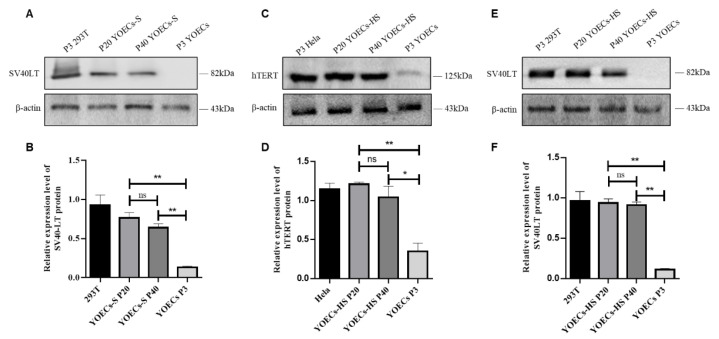
Expression stability of hTERT and SV40LT in immortalized YOECs. (**A**,**C**,**E**) Representative Western blots showing hTERT (125 kDa) and SV40LT (82 kDa) protein expression in YOECs-S (P20/P40) and YOECs-HS (P20/P40). (**B**,**D**,**F**) Densitometric quantification of band intensities normalized to β-actin (43 kDa) with corresponding statistical analysis. Data represent mean ± SD (n = 3); * *p* < 0.05; ** *p* < 0.01; ns: not significant. Primary YOECs (P3; negative control), HeLa (hTERT-positive), and 293T (SV40LT-positive) included. Molecular weight markers indicated in kDa.

Protein expression stability of hTERT (~125 kDa) and SV40LT (~82 kDa) was evaluated in immortalized YOECs-S and YOECs-HS cells at passages 20 (P20) and 40 (P40). Primary YOECs (P3) served as negative controls, while HeLa (hTERT-positive) and 293T (SV40LT-positive) cells provided positive controls. Western blot analysis revealed SV40LT expression exclusively in YOECs-S but absent in primary YOECs (Figure 6A,B). In contrast, both hTERT and SV40LT proteins were stably expressed in YOECs-HS, while primary YOECs exhibited basal hTERT expression. Critically, the expression levels of hTERT and SV40LT remained consistent across passages P20 to P40 in both YOECs-S and YOECs-HS cell lines, showing no significant changes (*p* > 0.05; Figure 6C–F). These results demonstrate stable protein expression characteristics in these immortalized cell lines during prolonged culture.

### 3.7. Absence of Malignant Characteristics in Immortalized YOECs

**Figure 7:**
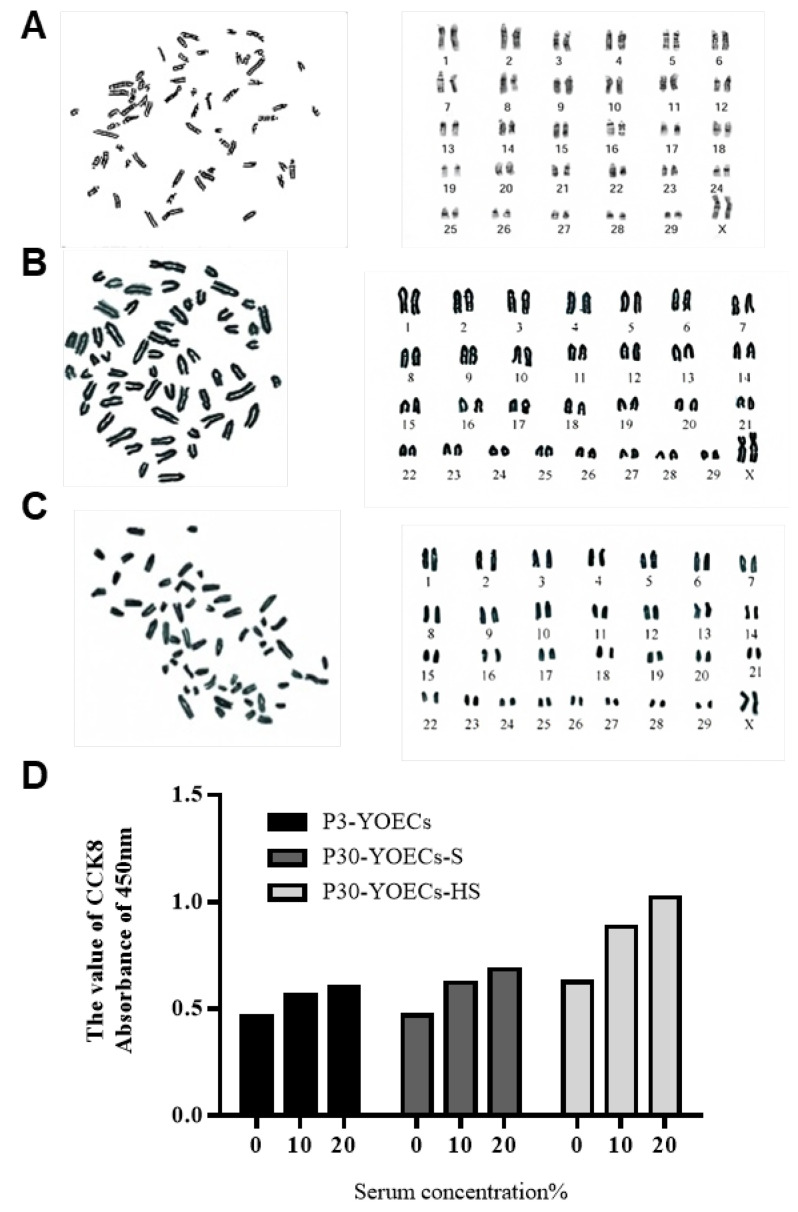
Karyotypic stability and serum dependence of immortalized YOECs. (**A**) Representative karyogram of primary YOECs (P3) showing normal diploid count (2n = 60). (**B**) Karyotype analysis of immortalized YOECs-S at P30. (**C**) Karyotype of immortalized YOECs-HS at P30. (**D**) Serum-dependence growth curves: cell viability at 72h across serum concentrations (0%, 10%, 20%). Data represent mean ± SD (n = 3).

Karyotype analysis revealed that immortalized YOECs-S and YOECs-HS maintained a normal diploid chromosome count of 60, consistent with primary YOECs (Figure 7A). No chromosomal aberrations (translocations, deletions, or aneuploidy) were detected, indicating preserved genomic integrity.

Furthermore, both immortalized lines exhibited serum-dependent growth kinetics indistinguishable from primary cells across serum concentration gradients (0%, 10%, 20%). All cell populations showed minimal proliferation under serum-free conditions, with dose-dependent increases in proliferative activity at 10% and 20% serum concentrations (Figure 7B). These findings demonstrate retention of normal growth regulation mechanisms without evidence of malignant transformation.

### 3.8. Functional Validation of Immortalized YOECs

**Figure 8:**
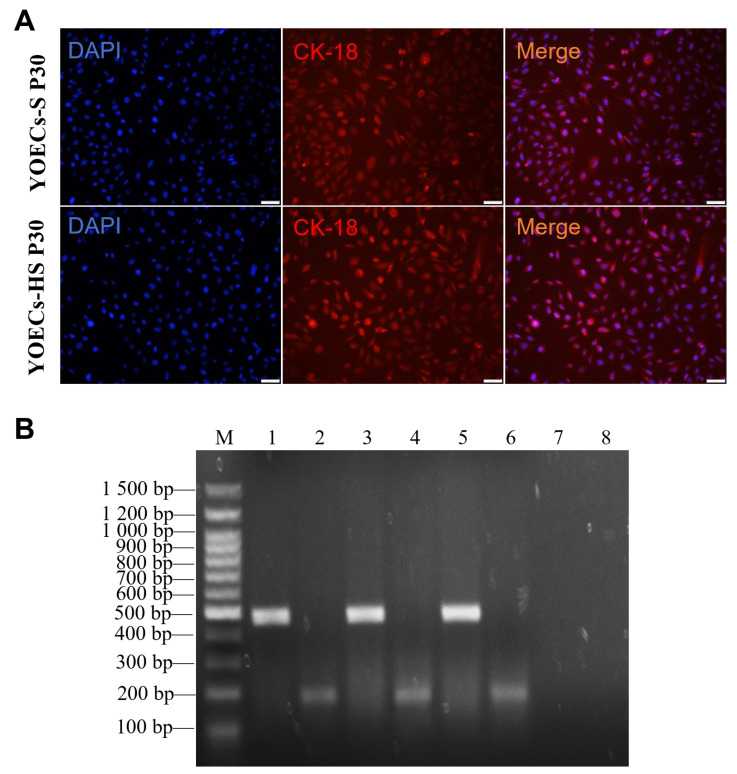
Functional characterization of immortalized YOECs. (**A**) Representative immunofluorescence images showing CK-18 expression (green) in P30 YOECs-S and YOECs-HS. Nuclei counterstained with DAPI (blue)(200×, Scale bar indicates 50 µm). (**B**) PCR detection of ERα and PR expression: Lane M: DNA marker; Lanes 1–2: Primary YOECs (P3); Lanes 3–4: YOECs-S (P30); Lanes 5–6: YOECs-HS (P30); Lanes 7–8: Blank controls.

Immortalized YOECs-S and YOECs-HS (P30) retained characteristic epithelial phenotypes, as confirmed by positive cytokeratin-18 (CK-18) immunofluorescence with intensity and distribution patterns indistinguishable from primary YOECs (Figure 8A). Both lines maintained stable expression of estrogen receptor α (ERα) and progesterone receptor (PR) at levels comparable to primary cells, as determined by PCR analysis (Figure 8B). These findings demonstrate preservation of epithelial identity and hormonal responsiveness in immortalized YOECs, indicating intact regulatory mechanisms for embryonic transport and immune microenvironment maintenance.

## 4. Discussion

We successfully established and characterized immortalized yak oviductal epithelial cell (YOEC) lines. Cells at passage 30 retained diploid karyotype, exhibited serum-dependent growth and contact inhibition, with stable expression of estrogen receptor (ER) and progesterone receptor (PR). Both cell lines (YOECs-S and YOECs-HS) have been stably propagated beyond passage 50 while maintaining consistent proliferation.

Bovine oviductal epithelial cells (BOECs) significantly enhance in vitro embryo development under co-culture conditions, particularly in hypoxic environments [21]. Primary YOECs were isolated through mechanical scraping coupled with collagenase digestion [22,23]. Immunofluorescence for cytokeratin-18 (CK-18; epithelial marker) [24,25] and vimentin (fibroblast marker) further validated their epithelial identity [26,27].

To achieve immortalization, we constructed lentiviral plasmids expressing hTERT (pLVX-hTERT-Puro) [28,29] and SV40LT (pLVX-SV40LT-Puro) [30,31]. YOECs-H ceased proliferation by passage 10, indicating that hTERT expression alone was insufficient for immortalization. This aligns with reports in Mongolian sheep OECs, where hTERT extended proliferative capacity but did not confer immortality. While hTERT counteracts replicative senescence by telomere maintenance [29], in vitro senescence can be driven by additional mechanisms—including oxidative stress, epigenetic drift, or tumor suppressor pathway activation [32,33]. SV40LT protein promotes proliferation by inactivating p53 and Rb tumor suppressors [34]. Notably, species-specific differences exist; murine cells readily immortalize with telomerase and p53 disruption, whereas human somatic cells typically require additional genetic alterations [35]. Hypoxia-adapted yak cells may exhibit unique stress responses; sustained HIF-1α activation could accelerate senescence in hTERT-only transduced YOECs [36]. Recent evidence confirms that hTERT often requires co-expression with immortalizing oncogenes (e.g., SV40LT) for complete immortalization [37], particularly in highly differentiated epithelial cells like YOECs.

BothYOECs-S (SV40LT alone) and YOECs-HS (hTERT + SV40LT) achieved immortalization (>P50). YOECs-HS exhibited superior growth characteristics and genomic stability. This highlights the synergistic role of hTERT and SV40LT: while SV40LT overcomes cell-cycle arrest by suppressing p53/Rb pathways [38,39], its high expression alone may induce metabolic dysregulation and proto-oncogene activation (e.g., c-Myc). In YOECs-HS, hTERT alleviates telomere-dependent senescence, reducing the required SV40LT expression level. This balance enables sustained proliferation while minimizing transformation risk. While SV40LT is essential for YOEC immortalization, optimal stability requires hTERT co-expression. Exogenous hTERT prevents telomere attrition, enabling unlimited proliferation while preserving physiological properties [38]. These stable lines overcome primary cell-passaging limitations and provide a much-needed in vitro platform for high-altitude reproductive research.

To guarantee its reliability in complex physiological studies, we have instituted a phased verification program and outlined the following prospective work. At the current stage (P30), we have completed molecular validation of immortalization (robust telomerase activity and stable SV40LT expression) and basic phenotypic stability assessments (growth kinetics and karyotype analysis), confirming expression of fundamental epithelial markers (CK-18+/Vimentin-). In subsequent work (≥P50), we will systematically quantify functional markers—ciliation (FOXJ1/α-tubulin) [40], secretory phenotype (OVGP1, LCN2) [41], and barrier integrity (Occludin/Claudins) [42]—and establish an embryo–oviduct co-culture model. Yak embryos are indirectly co-cultured with YOEC-HS in a Transwell system to evaluate effects on embryonic development rates. Secreted embryotrophic factors (e.g., miRNAs and metabolites) will be profiled via exosome isolation and multi-omics analyses. To mitigate SV40LT-related risks, we will continuously monitor oncogene expression (e.g., c-Myc) and institute an early-warning passage threshold (e.g., P80). If ciliation frequency or OVGP1 secretion declines by >30%, a fresh batch of cells will be thawed to ensure experimental reproducibility.

Beyond serving as a foundational in vitro tool, the YOEC-HS line holds significant promise for advancing yak reproductive biotechnology. Its availability enables species-specific investigation into high-altitude adaptive mechanisms governing embryo–maternal dialog—a domain previously inaccessible due to the scarcity of primary yak oviductal tissue. Crucially, by modeling the oviductal microenvironment through planned co-culture systems, this platform may directly inform the optimization of IVP protocols. These protocols were tailored to yak physiology, thereby addressing the pressing issue of low reproductive efficiency exacerbated by genetic deterioration. Furthermore, our proactive quality control framework—monitoring oncogenic risks and functional stability beyond P50—ensures the reliability required for long-term mechanistic studies. While challenges regarding epigenetic drift in vitro versus in vivo functionality remain, this immortalized model represents a critical step toward deciphering the unique reproductive ecology of plateau yaks and developing targeted interventions for conservation and breeding.

## 5. Conclusions

We established an optimized protocol for primary yak oviduct epithelial cell (YOEC) isolation and culture, subsequently generating immortalized lines via distinct lentiviral-mediated approaches. Both YOECs-S (SV40LT-only) and YOECs-HS (dual SV40LT/hTERT) exhibit enhanced proliferative capacity, stable transgene expression, intact hormonal responsiveness (ERα/PR retention), and non-transformed phenotypes (diploid karyotype, serum dependence). These lines have maintained consistent characteristics through >50 passages, providing validated in vitro models for optimizing yak embryo–oviduct epithelial co-culture systems.

## Figures and Tables

**Table 1:** Primer Information.

Gene Name	Primer Sequence
*hTERT*	F: CGTGGTTTCTGTGTGGTGTC
	R: CCTTGTCGCCTGAGGAGTAG
*SV40LT*	F: ATTGCCTGGAACGCAGTGA
	R: GCAAACTCAGCCACAGGTCT
*GAPDH*	F: GAAGGTCGGAGTGAACGGA
	R: CTTGCCGTGGGTGGAATCAT
*ERα*	F: TACGGAAAGACCGAAGAGGAG
	R: CCAGGAGAAGGTTAGGAGCAA
*PR*	F: TCAACTACCTGAGGCCGGAT
	R: ACTTTCGGCCTTCCATTGC

ER: Estrogen receptor; PR: Progesterone receptor.

## Data Availability

All data presented in this study are available on request from the corresponding authors.
